# Genetic features of B‐cell lymphoblastic lymphoma with *TCF3‐PBX1*

**DOI:** 10.1002/cnr2.1559

**Published:** 2021-09-23

**Authors:** Ryota Shirai, Tomoo Osumi, Aiko Sato‐Otsubo, Kazuhiko Nakabayashi, Takeshi Mori, Masanori Yoshida, Kaoru Yoshida, Mika Kohri, Takashi Ishihara, Shiho Yasue, Toshihiko Imamura, Mikiya Endo, Satoshi Miyamoto, Kentaro Ohki, Masashi Sanada, Nobutaka Kiyokawa, Seishi Ogawa, Takako Yoshioka, Kenichiro Hata, Masatoshi Takagi, Motohiro Kato

**Affiliations:** ^1^ Department of Pediatric Hematology and Oncology Research National Research Institute for Child Health and Development Tokyo Japan; ^2^ Department of Pediatrics Yokohama City University Graduate School of Medicine Yokohama Japan; ^3^ Children's Cancer Center National Center for Child Health and Development Tokyo Japan; ^4^ Department of Maternal‐Fetal Biology National Research Institute for Child Health and Development Tokyo Japan; ^5^ Department of Hematology and Oncology Hyogo Prefectural Kobe children's Hospital Kobe Japan; ^6^ Department of Hematology, Comprehensive Cancer Center, International Medical Center Saitama Medical University Saitama Japan; ^7^ Department of Pediatrics Nara Medical University Kashihara Japan; ^8^ Department of Pediatrics, Graduate School of Medicine Gifu University Gifu Japan; ^9^ Department of Pediatrics Kyoto Prefectural University of Medicine, Graduate School of Medical Science Kyoto Japan; ^10^ Department of Pediatrics Iwate Medical University Morioka Japan; ^11^ Department of Pediatrics and Developmental Biology Tokyo Medical and Dental University Tokyo Japan; ^12^ Clinical Research Center National Hospital Organization Nagoya Medical Center Nagoya Japan; ^13^ Department of Pathology and Tumor Biology, Graduate School of Medicine Kyoto University Kyoto Japan; ^14^ Department of Pathology National Center for Child Health and Development Tokyo Japan

**Keywords:** 6q LOH, B‐cell lymphoblastic lymphoma, *KMT2D*, *TCF3‐PBX1*, whole exome sequencing

## Abstract

**Background:**

Lymphoblastic lymphoma (LBL) and acute lymphoblastic leukemia (ALL) are categorized as the same entity under precursor lymphoid neoplasms in the World Health Organization classification. However, compared to B‐cell ALL, the molecular genetic makeup of B‐cell LBL remains to be understood, mainly due to its rarity. We performed whole exome sequencing (WES) on seven patients with *TCF3‐PBX1*‐positive B‐cell LBL.

**Methods:**

WES was performed using DNA extracted from tumor specimens and paired blood samples at remission for six patients, and tumor‐only analysis was performed for one patient whose remission sample was not available. For one patient, a relapsed sample was also analyzed.

**Results:**

*KMT2D* variants and 6q LOH were found as recurrent alterations. Somatic variants of *KMT2D* were identified in three of the seven patients. Of note, the two patients with heterozygous nonsense variant of *KMT2D* were at stage III, without bone marrow infiltration. 6q LOH was also identified in two others, out of the seven patients. The common 6q deleted region of the two patients ranged from 6q12 to 6q16.3. Both patients had bone marrow infiltration. Analysis of recurrent case also revealed that the relapsed clone might be derived from a minor clone of the bone marrow at diagnosis.

**Conclusion:**

In this study, through WES for seven patients with *TCF3‐PBX1*‐positive B‐LBL, we identified *KMT2D* mutations and 6q LOH as recurrent alterations. In order to elucidate the relationship between these recurrent alterations and disease specificity or outcomes, further studies comparing with *TCF3‐PBX1*‐positive B‐ALL are required.

## 1 INTRODUCTION

Lymphoblastic lymphoma (LBL) and acute lymphoblastic leukemia (ALL) are categorized as the same entity under precursor lymphoid neoplasms in the World Health Organization classification.^1^ Recent advances in genetic analysis technology revealed the genomic landscape of ALL, which contributes not only to understanding pathogenesis, but also to improvements in treatment outcome. However, in spite of the resemblance in morphological and immunophenotypic features between ALL and LBL, the molecular genetic makeup of LBL remains to be understood, particularly for B‐cell LBL, mainly due to its rarity.

Recently, our group reported a case series of *TCF3‐PBX1*‐positive B‐LBL,^2^ which suggested the potentially poor outcomes of *TCF3‐PBX1*‐positive LBL. Previous studies in mice have shown that *TCF3‐PBX1* needs to cooperate with additional gene alterations to develop B‐ALL.^3^, ^4^ We performed whole exome sequencing (WES) to characterize genetic alterations in seven patients with *TCF3‐PBX1*‐positive B‐LBL, including five patients described in our previous report and two additional patients.

## 2 MATERIALS AND METHODS

### 2.1 Patients and samples

This study was approved by the Ethics Committee at the National Center for Child Health and Development (#1035), and the required written informed consent was obtained from the parents and/or guardians. The *TCF3‐PBX1* fusion was confirmed by the evidence of t(1;19)(q23;p13.3), reverse transcriptase PCR,^5^ fluorescent in situ hybridization (FISH),^6^ or RNA sequencing (Table S1). Bone marrow aspiration was performed from one side of the pelvic bones and bone marrow was evaluated microscopically by pathologists. Seven patients with the diagnosis of B‐LBL with *TCF3‐PBX1* between 2013 and 2018 were enrolled retrospectively. WES was performed using DNA extracted from tumor specimens at diagnosis and paired blood samples at remission for six patients, and tumor‐only analysis was performed for one patient whose remission sample was not available. For one patient, a relapsed sample was also analyzed.

### 2.2 Whole exome sequencing

DNA was extracted using the QIAamp DNA Mini Kit or GeneRead DNA FFPE Kit (Quiagen, Hilden, Germany) following the manufacturer's instructions. After fragmentation of the DNA to approximately 200 bp with advanced focused acoustics (Covaris), library construction was performed with a combination of SureSelect HumanAll Exon Kit (Agilent Technology) and KAPA Hyper Prep Kit (NIPPON Genetics) according to the manufacturers' protocols. Enriched fragment libraries were then sequenced on an Illumina HiSeq 2500 in 101‐bp paired‐end mode. A robust bioinformatics pipeline validated previously was used for alignment, variant calling, and analyzing copy number variation (Supporting Information Methods).

## 3 RESULTS

The genomic and clinical features for seven patients are summarized in Table 1 and Table S1. The median age at diagnosis was 13 years (range 5–16 years). Two patients were at stage III according to the St. Jude classification for pediatric non‐Hodgkin lymphoma (NHL).^7^ Five patients were at stage IV with bone marrow involvement by molecular methods as defined by minimal disseminated disease (MDD). Although the number of blast cells in bone marrow was microscopically in the normal range in five cases, minimal disseminated disease in bone marrow was positive in four out of four evaluated cases by PCR or FISH for *TCF3‐PBX1*. The NHL‐BFM95‐based chemotherapeutic regimen^8^ was used for all patients. Two of the seven patients relapsed. UPN2 relapsed at thorax immediately after termination of maintenance therapy and was treated for hematopoietic cell transplantation. UPN4 relapsed as a leukemia and abnormal accumulation on gallium scintigraphy in the left third rib was also found and died because of tumor progression after receiving hematopoietic cell transplantation.

**TABLE 1:** Clinical characteristics and recurrent genomic alterations

UPN	Age (years)	Sex	Location of tumor	Blasts in BM (%)	MDD in BM	Stage	Outcome (follow‐up duration after CR, months)	*KMT2D*	1q gain	6q LOH
1	16	M	Nasal cavity^a^	0	N.E.	III	CR1^§^	c.8401C > T (p.Arg2801Ter)	Yes	No
2	15	F	Femur^a^, ovary, thorax	0	N.E.	III	CR2^22^	c.16360C > T (p.Arg5454Ter)	Yes	No
3	5	F	Head^b^, bilateral kidney	19.2	PCR (+)	IV	CR1^c^	c.4344 T > G (p.Cys1448Trp)	Yes	No
4	14	M	Palate^b^, jaw	16	N.E.	IV	DOD		Yes	Yes
5	7	F	Tibia^b^, pancreas, kidney	0	PCR (+)	IV	CR1^c^		Yes	Yes
6	13	F	Pancreas^b^, thoracic vertebrae	1	FCM (+)	IV	CR1 (46)		No	No
7	9	F	Extracranial tissue^a^, jaw, ilium, lumbar vertebrae, rib, kidney	1.2	PCR (+)	IV	CR1 (84)		No	No

Abbreviations: BM, bone marrow; CR, complete remission; DOD, dead of disease; F, female; FCM, flow cytometry; HSCT, hematopoietic stem cell transplantation; LOH, loss of heterozygosity; M, male; MDD, minimal disseminated disease; N.E., not examined; PCR, polymerase chain reaction.

^a^ Formalin‐fixed, paraffin‐embedded (FFPE) tissue was used for tumor genetic testing.

^b^ Fresh frozen tissue was used for genetic analysis.

^c^ These patients are on therapy.

Somatic variants of *KMT2D* were identified in three of the seven patients. UPN1 and UPN2 had heterozygous nonsense variants of c.8401 T > C (p.Arg2801Ter) and c.16360C > T (p.Arg5454Ter) in the SET domain of the *KMT2D* gene, respectively (Table S2 and Figures S1 and S2), which had been known as being pathogenic.^9^ Of note, the two patients were at stage III, without bone marrow infiltration. UPN3 had a novel heterozygous missense variant of c.4344 T > G (p.Cys1448Trp) in the PHD domain of the *KMT2D* gene.

**FIGURE 1:**
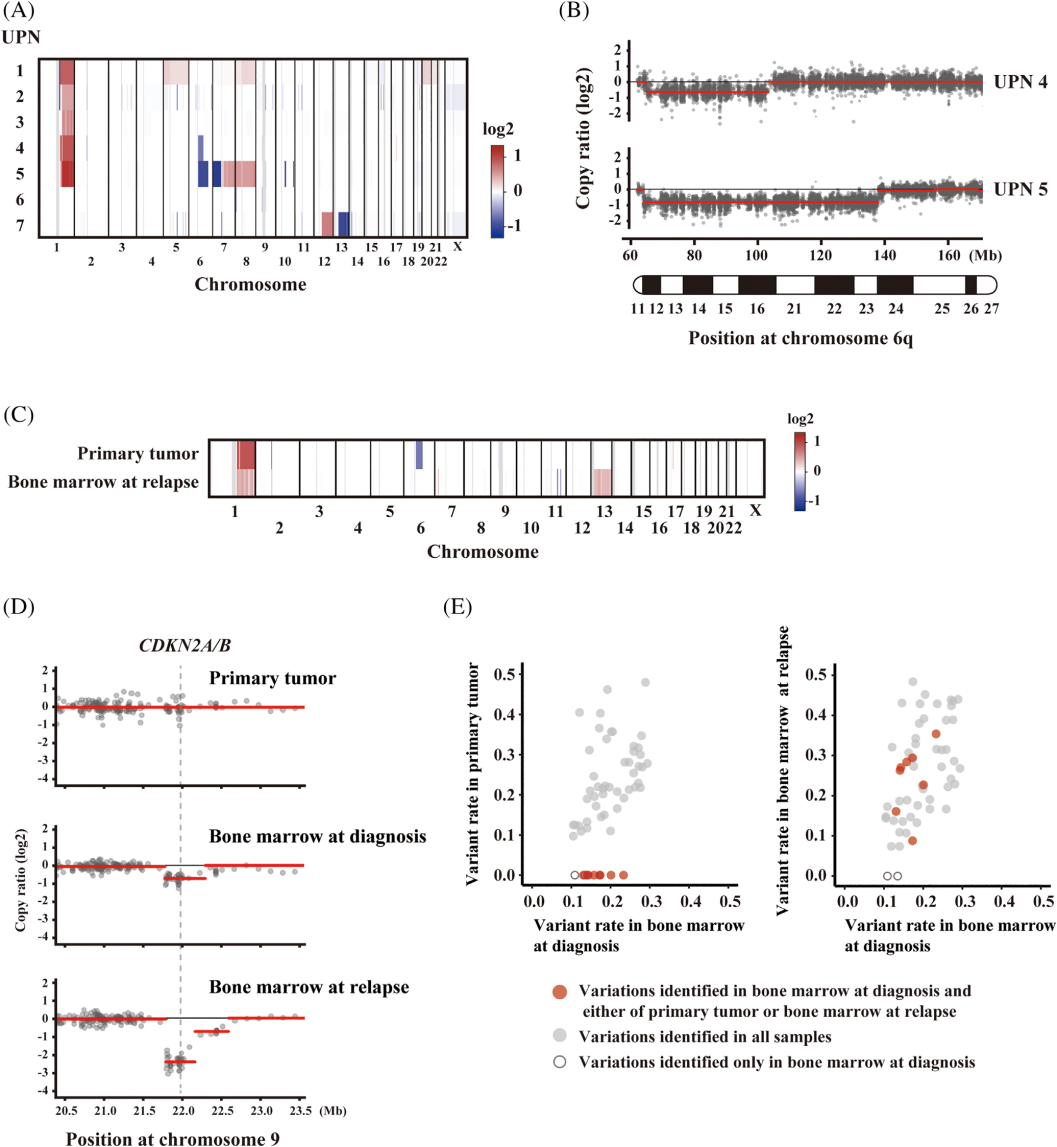
Genomic copy number alterations in the tumor specimen. Genomic copy number alterations were identified using whole‐exome sequencing followed by copy number calling. (A) A heatmap of the copy number ratio for each tumor specimen at diagnosis. The values for all inferred segments are depicted by the color indicated on the right. (B) Scatter plots of the copy number ratio on 6q are shown. Each dot indicates the value for the targeted region. Red lines represent the copy number segment inferred in the analysis. All positions shown in this figure were based on GRCh37. (C), (E) Comparison of genomic alterations between diagnostic and relapsed specimens. (C) A heatmap of the genomic copy number alterations in primary tumor and bone marrow at relapse in UPN4. All the inferred segments are color‐coded by the indicated color according to their copy number ratio. (D) Scatter plots of the copy number ratio around *CDKN2A/B* loci. Each dot indicates the value for the targeted region. Red lines represent the copy number segment inferred in the analysis. All the positions shown in this figure were based on GRCh37. (E) Scatter plots of the rate of the single nucleotide variants are shown, in which each dot indicates the variants identified in the bone marrow specimen taken at initial diagnosis. In each panel, variant rates were compared between bone marrow at diagnosis and primary tumor at diagnosis (left side) or bone marrow at relapse (right side), respectively. Dots on the *x*‐axis indicate that any sequence read was not detected, or that the variant rate was 0%, in the tumor specimen

Copy number analysis revealed a gain of chromosome 1q and a loss of heterozygosity at the long arm of chromosome 6 (6q LOH) as recurrent alterations (Figure 1A and Table S3). The 1q gain was detected in five of the seven patients. It may reflect an unbalanced translocation between chromosome 1q23 (*PBX1*) and 19p13 (*TCF3*), which accounts for 75% in *TCF3‐PBX1*‐positive B‐ALL.^10^ 6q LOH was also identified in two others, out of the seven patients. The common 6q deleted region of the two patients ranged from 6q12 to 6q16.3 (Figure 1B and Table S3). Both patients had bone marrow infiltration.

We then focused on the genomic features of the relapsed specimen (UPN4).

WES showed that additional genetic hits were discordant between relapsed and primary tumors. The 6q LOH, which was identified in the primary tumor specimen, was not detected in the relapsed bone marrow, whereas gain of 13q and loss of 9p, 11q, 15q, and 17p, which were identified in the relapsed specimen, were not detected in the primary tumor specimen (Figure 1C and Table S4). By contrast, a deletion of *CDKN2A/B* on 9p in the relapsed bone marrow was identified in the primary bone marrow as a minor clone population, but not in the primary tumor specimen (Figure 1D). Whereas some of the relapse‐specific single nucleotide variants were also detected in the bone marrow at diagnosis, they were not detected in the primary tumor specimen (Figure 1E). These findings revealed that the relapsed clone might be derived from a minor clone of the bone marrow at diagnosis (Figure S3).

## 4 DISCUSSION

Unlike ALL, the majority of LBL is T‐lymphoblastic in origin, and B‐LBL consists of only 20%–25% of pediatric LBL cases. Due to the rarity and limited availability of tumor samples, genetic features of B‐LBL are almost unknown. In this study, through WES for seven patients with *TCF3‐PBX1*‐positive B‐LBL, we identified *KMT2D* mutations and 6q LOH as recurrent alterations.

The *KMT2D* gene, also known as *MLL2*/*MLL4*, encodes a histone H3 lysine 4 (H3K4) mono‐methyltransferase on enhancer regions and plays a crucial role in maintaining genomic stability.^11^ Previous reports demonstrated that the *KMT2D* gene acts as a tumor suppressor in various cancers.^12^ In pediatric B‐ALL, *KMT2D* mutation was found in 6.2%, which was the most frequently mutated epigenetic regulator and is considered to be the driver gene.^13^, ^14^, ^15^ Ueno H et al. also reported that frequent involvement of *KMT2D* mutations were one of characteristic of *TCF3‐PBX1*‐positive B‐ALL and was a poor prognosis factor for B‐ALL.^16^

Two of the three somatic mutations detected in our study were the nonsense mutations affecting the catalytic SET domain, which is responsible for H3K4 methyltransferase activity and maintaining KMT2D protein stability (Figure S2).^9^ Another is the missense mutation that is located in the PHD domain, which recognize H4 tails on nucleosomes and could be critical for KMT2D‐catalyzed nucleosome methylation.^17^ The missense variants in the PHD domain have been reported in various tumors,^18^, ^19^, ^20^ suggesting an important carcinogenic role as a tumor suppressor.^9^ *KMT2D* mutations might cooperate with *TCF3‐PBX1* to develop lymphoid tumor, but it is unclear why they were lymphomas. Of note, the patients with nonsense variants of the *KMT2D* had no bone marrow infiltration, which suggests that dysfunction of *KMT2D* might contribute to the lymphomatous features of LBL, however further study is needed to clarify it.

Previous studies showed that 6q LOH occurred recurrently in lymphoid neoplasms in up to 9.0% of pediatric ALL cases, although its impact on outcomes was unclear unlike T‐LBL with 6q LOH, which is associated with unfavorable prognosis.^21^, ^22^ The common deleted region in our two patients overlapped the deleted region reported in cases of T‐LBL and ALL.^22^, ^23^ In T‐LBL or T‐ALL, *GRIK2* (6q16.3), *CASP8AP2* (6q15‐16.1), and *EPHA7* (6q16.1) were reported as candidates for tumor suppressor genes in this deletion region, but it is unclear whether they are involved in the difference between LBL and ALL.^24^, ^25^, ^26^

This study has several limitations. First, due to the small sample size and the short observation period, the precise prevalence of recurrent genomic alterations and the correlation between clinical features and those variants were unable to be accurately assessed. Second, our copy number analysis may have been underestimated due to the variability in the exon capture procedure and not evaluating the intron area.

Ueno H et al. recently published on the landscape of B‐ALL,^27^ and found significant correlation between *TCF3‐PBX1* subtype and alterations in *RB1*, *PAX5*, and *PHF6*. It is interesting that UPN3 who was involved bone marrow had *PHF6* mutation besides *KMT2D* mutation (Supporting Information Table S2). In conclusion, we found *KMT2D* variants and 6q LOH as recurrent alterations in *TCF3‐PBX1*‐positive B‐LBL. In order to elucidate the relationship between these recurrent alterations and disease specificity or outcomes, further studies with additional cases comparing with *TCF3‐PBX1*‐positive B‐ALL are required.

## CONFLICT OF INTEREST

We confirm that we have no financial conflicts of interest concerning the publication of this manuscript.

### AUTHOR CONTRIBUTIONS

**R.S.**: Formal analysis (equal); validation (lead); visualization (equal); writing – original draft (lead). **T.O.**: Conceptualization (equal); data curation (lead); resources (lead); supervision (equal). **A.S.**: Formal analysis (lead); visualization (equal); writing – original draft (equal). **K.N.**: Formal analysis (equal); software (equal). **T.M.**: Data curation (equal). **M.Y.**: Formal analysis (equal); software (equal). **K.Y.**: Data curation (equal). **M.K.**: Resources (equal). **T.I.**: Resources (equal). **S.Y.**: Resources (equal). **T.I.**: Resources (equal). **M.E.**: Resources (equal). **S.M.**: Data curation (equal); resources (equal). **K.O.**: Supervision (equal). **M.S.**: Data curation (equal); supervision (equal). **N.K.**: Supervision (equal). **S.O.**: Software (equal); supervision (equal). **T.Y.**: Data curation (equal); resources (equal). **K.H.**: Formal analysis (equal); software (equal). **M.T.**: Conceptualization (equal); data curation (equal); resources (equal); supervision (equal). **M.K.**: Conceptualization (equal); funding acquisition (lead); methodology (lead); project administration (lead); supervision (lead); writing – review and editing (lead).

## ETHICS STATEMENT

This study was approved by the Ethics Committee at the National Center for Child Health and Development (#1035), and the required written informed consent was obtained from the parents and/or guardians.

## Supporting information


**Appendix S1**: Supporting Information

## Data Availability

The data that support the findings of this study are available from the corresponding author upon reasonable request.
