# Supplementary material for: Genetic features of B‐cell lymphoblastic lymphoma with TCF3‐PBX1
Source: Cancer Rep (Hoboken). 2021 Sep 23;5(9):e1559. doi: 10.1002/cnr2.1559 (PMC9458492; doi:10.1002/cnr2.1559)
Supplement: Supplementary file 1 — Appendix S1: Supporting Information [file CNR2-5-e1559-s001.pdf]

Supporting Information

Supporting Information Methods

Bioinformatic analysis

Paired-end reads obtained from whole-exome sequencing were aligned to the reference human genome (GRCh37) using bwa (version 0.7.8) implemented in Genomon pipeline (version 2.6.3). Somatic mutations were also explored using Genomon pipeline, where EBCall was performed with default settings. Mutations with (i) *p*-value of Fisher's exact test <0.01, (ii) those of EBCall[1] <0.0001, (iii) variant allele frequency >0.1 in tumor sample, and (iv) whose sequencing depth of the tumor sample >10 were considered as candidate variants. Variants located in segmental duplication regions annotated according to GenomicSuperDups table (version hg19) were removed from the candidate list.

Copy number analysis

Copy number status was inferred using CNVkit[2] (version 0.9.6), in which the number of mapped reads was calculated for each targeted region, then the values of tumor genome were compared with those of reference normal samples. Copy number segmentation was done using circular binary segmentation (CBS) method with default parameter settings. Log2 ratios >0.2 and <-0.2 presented in a continuous range of 20 Mb or more were

assigned as the threshold for gain and loss detection. Gap lesions up to 10 kb in each segment were acceptable. Copy number ratio of X chromosome were adjusted to the expected number of X chromosome, based on the patient's sex.

Bioinformatic analysis of the relapsed case

In order to compare the differences in single nucleotide variants between the specimens at diagnosis and relapse, more rigorous criteria were used as follows : (i) *p*-values of EBCall <0.01, (ii) variant allele frequencies >0.1, (iii) whose sequencing depths of all three specimen taken from this patient >50, and (iv) not located in segmental duplication regions in GenomicSuperDups table (version hg19). For excluding the SNPs, candidates in the dbSNP 138 database NonFlagged set were also excluded, as well as whose variant rates in primary BM specimen were large (>=0.3) as the tumor rate was 16%.

Copy number analysis of the relapsed case

In order to compare the differences between the specimens at the diagnosis and the relapse, more rigorous and reliable criteria were used. Log2 ratios > 0.4 and < -0.4 including 40 probes or more were assigned as the threshold for gain and loss. Gap lesions up to 10 kb in each segment were acceptable. Immunoglobulin gene rearrangements and copy

number alterations in the same gene region between tumor specimens at diagnosis and relapse were manually excluded.

Supporting Information References

1. Shiraishi Y, Sato Y, Chiba K, et al. An empirical Bayesian framework for somatic mutation detection from cancer genome sequencing data. *Nucleic acids research.* 2013;41(7):e89.
2. Talevich E, Shain AH, Botton T, Bastian BC. CNVkit: Genome-Wide Copy Number Detection and Visualization from Targeted DNA Sequencing. *PLoS computational biology.* 2016;12(4):e1004873.

TABLE S1 Additional sample information

UPN	Karyotype analysis	Fusion FISH analyses	Test to confirm *TCF3-PBX1*	Immunohistochemical staining	Flow cytometry analysis
1	50,XY,t(1;19)(q23;p13.3),+4,+5,+8, der(15),t(1;15)(q12:p11.2),-19, +12,+20	Not examined	PCR	CD19+, CD20+, TdT+, CD3-, MPO-	CD10+, CD19+, CD20+, CD38+, IgM+, λ -chain+, CD3-, CD5-, CD25-
2	46,XX,add(5)(q11.2),der(19)t(1;19)(q23;p13.3)	Not examined	PCR	CD10+, CD79+, Ki67+, TdT+	CD10+, CD19+, CD38+, CD3-, CD20-
3	A:46,XX,inv(9)(p12q13) [18/20] B: 45,XX,-6,-9,-13, der(19)?t(1;19)(q23;p13.3), +der(?)t(?;1)(?;q21),mar1 [2/20]	Positive	PCR	CD10+, CD19+, CD24+, cytoplasmic μ chain+	CD19+, CD22+, cyCD79a+, cytoplasmic μ chain+
4	46,XY,+1,der(1;15)(q10;q10), t(1;19)(q23;p13) [2] 46,idem,-9,+r1 [2] 46,XY [16]	Not examined	PCR	No data	CD10+, CD19+, CD3-, CD20-
5	46,XX	Not examined	PCR	CD10+, CD79a+, TdT+, CD3-, CD20-, CD5-, CD23-	CD10+, CD19+, CD3-, CD20-
6	No mitotic phase available	Positive	PCR, RNAseq	CD 10+, CD19+, TdT+, CD3-, CD20-, MPO-	CD10+, CD19+, CD38+, CD3-, CD20-
7	Not examined	Positive	PCR	CD20 (-/+), CD79a+, TdT+	No data

FISH, fluorescent in situ hybridization; PCR, polymerase chain reaction; RNAseq, RNA sequencing analysis; MPO, myeloperoxidase; TdT, terminal deoxynucleotidyl transferase

TABLE S2 Non-silent variants identified by whole-exome sequencing

Case	**Gene**	**Cytoband**	**Position**	**Annotation**	**Allele frequency**	**-log10 (P-value) (Fisher)**
UPN1	*EXPH5*	11q22.3	chr11:108380462	c.T5772A:p.D1924E:NM_015065	0.17	3.56
UPN1	*HEPACAM2*	7q21.3	chr7:92848687	c.G121A:p.V41I:NM_198151	0.26	11.16
**UPN1**	***KMT2D***	**12q13.12**	**chr12:49432738**	**c.C8401T:p.R2801X:NM_003482**	**0.21**	**13.22**
UPN1	*NTRK3*	15q25.3	chr15:88671959	c.C1211T:p.T404M:NM_002530	0.19	2.16
UPN1	*OR2W3*	1q44	chr1:248059777	c.G889A:p.V297M:NM_001001957	0.35	16.40
UPN1	*TCEA1*	8q11.23	chr8:54891676	c.T734C:p.I245T:NM_006756	0.52	12.89
UPN2	*C8orf44*	8q13.1	chr8:67590151	c.C208T:p.R70X:NM_019607	0.31	2.11
UPN2	*KLHDC4*	16q24.2	chr16:87743248	c.G1070A:p.R357H:NM_017566	0.44	24.43
**UPN2**	***KMT2D***	**12q13.12**	**chr12:49416115**	**c.C16360T:p.R5454X:NM_003482**	**0.49**	**13.11**
UPN2	*NADSYN1*	11q13.4	chr11:71191918	c.G991A:p.E331K:NM_018161	0.41	9.25
UPN2	*NCAPH*	2q11.2	chr2:97024923	c.G1349A:p.R450Q:NM_015341	0.42	12.88
UPN2	*PDCD11*	10q24.33	chr10:105205151	c.G5461C:p.V1821L:NM_014976	0.48	23.07
UPN2	*UPF1*	19p13.11	chr19:18967769	c.C1908G:p.D636E:NM_002911	0.37	6.99
UPN2	*WWC2*	4q35.1	chr4:184182577	c.G1801A:p.E601K:NM_024949	0.39	13.26
UPN3	*CDC25B*	20p13	chr20:3782974	c.A1103T:p.E368V:NM_004358	0.10	2.44
UPN3	*CDH20*	18q21.33	chr18:59217418	c.G1856A:p.R619Q:NM_031891	0.37	9.48
**UPN3**	***KMT2D***	**12q13.12**	**chr12:49440466**	**c.T4344G:p.C1448W:NM_003482**	**0.43**	**15.02**
UPN3	*PHF6*	Xq26.2	chrX:133527551	c.T261A:p.C87X:NM_032335	0.52	20.20
UPN4	*ZCRB1*	12q12	chr12:42711695	c.C119G:p.T40S:NM_033114	0.43	3.69

UPN5	*C12orf56*	12q14.2	chr12:64678531	c.C743T:p.A248V:NM_001099676	0.34	5.41
UPN5	*CFAP69*	7q21.13	chr7:89917557	c.G1666A:p.G556R:NM_001039706	0.30	6.78
UPN5	*NRAS*	1p13.2	chr1:115258744	c.G38A:p.G13D:NM_002524	0.36	9.87
UPN5	*RBPJL*	20q13.12	chr20:43942711	c.G794A:p.R265Q:NM_014276	0.58	34.59
UPN7	*PODN*	1p32.3	chr1:53546494	c.C1751T:p.A584V:NM_153703	0.32	11.68
UPN7	*SV2A*	1q21.2	chr1:149885237	c.G156T:p.E52D:NM_014849	0.41	20.91
UPN7	*TRPC7*	5q31.1	chr5:135692424	c.C652T:p.R218C:NM_020389	0.46	25.30

Bold characters are recurrent variants of 7 cases. In UPN4, bone marrow at diagnosis was used for comparison. All positions shown in this figure were based on GRCh37.

TABLE S3 Genomic copy number alterations

Case	Arm	Gain / Loss	Position
**UPN1**	**1q**	**Gain**	**chr1:148890178-249231296**
UPN1	5p	Gain	chr5:10500-45907753
UPN1	5q	Gain	chr5:49555074-180899478
UPN1	8p	Gain	chr8:14034-43398986
UPN1	8q	Gain	chr8:47458041-86555432
UPN1	8q	Gain	chr8:87447641-146303522
UPN1	20p	Gain	chr20:68259-26256532
UPN1	20q	Gain	chr20:29420069-62965020
UPN1	21q	Gain	chr21:14300000-48111185
**UPN2**	**1q**	**Gain**	**chr1:164761536-249231296**
**UPN3**	**1q**	**Gain**	**chr1:164560355-248755453**
**UPN4**	**1q**	**Gain**	**chr1:148328155-249231296**
**UPN4**	**6q**	**Loss**	**chr6:65146064-103396980**
**UPN5**	**1q**	**Gain**	**chr1:148328757-249231296**
UPN5	7q	Gain	chr7:63213980-100331103
UPN5	7q	Gain	chr7:100344152-159128163
UPN5	8p	Gain	chr8:14034-43398986
UPN5	8q	Gain	chr8:47458041-146303522
**UPN5**	**6q**	**Loss**	**chr6:63990650-137941083**
UPN5	7p	Loss	chr7:13744-57938684
UPN7	12q	Gain	chr12:53413779-133841395
UPN7	13q	Loss	chr13:40174913-86759824
UPN7	13q	Loss	chr13:86910824-115109378

Only autosomal chromosomal alterations are shown. Bold characters are recurrent copy number alterations of 7 cases. All positions shown in this figure were based on GRCh37.

TABLE S4 Differences of genomic copy number alterations between primary and relapsed specimens in UPN4

Source	Arm	Gain/Loss	position (hg19)
Primary tumor at diagnosis	6q	Loss	65146064-103396980
Bone marrow at relapse	11q	Loss	85780359-88561596<br>103306620-105483169
Bone marrow at relapse	13q	Gain	19020500-86759824<br>86910824-115109378
Bone marrow at relapse	15q	Loss	30249213-30845207<br>32404338-32896222
Bone marrow at relapse	17p	Loss	15164631-15928696
Bone marrow at relapse	9p	Loss	21803390-22607426

Only autosomal chromosomal alterations are shown. All positions shown in this figure were based on GRCh37.

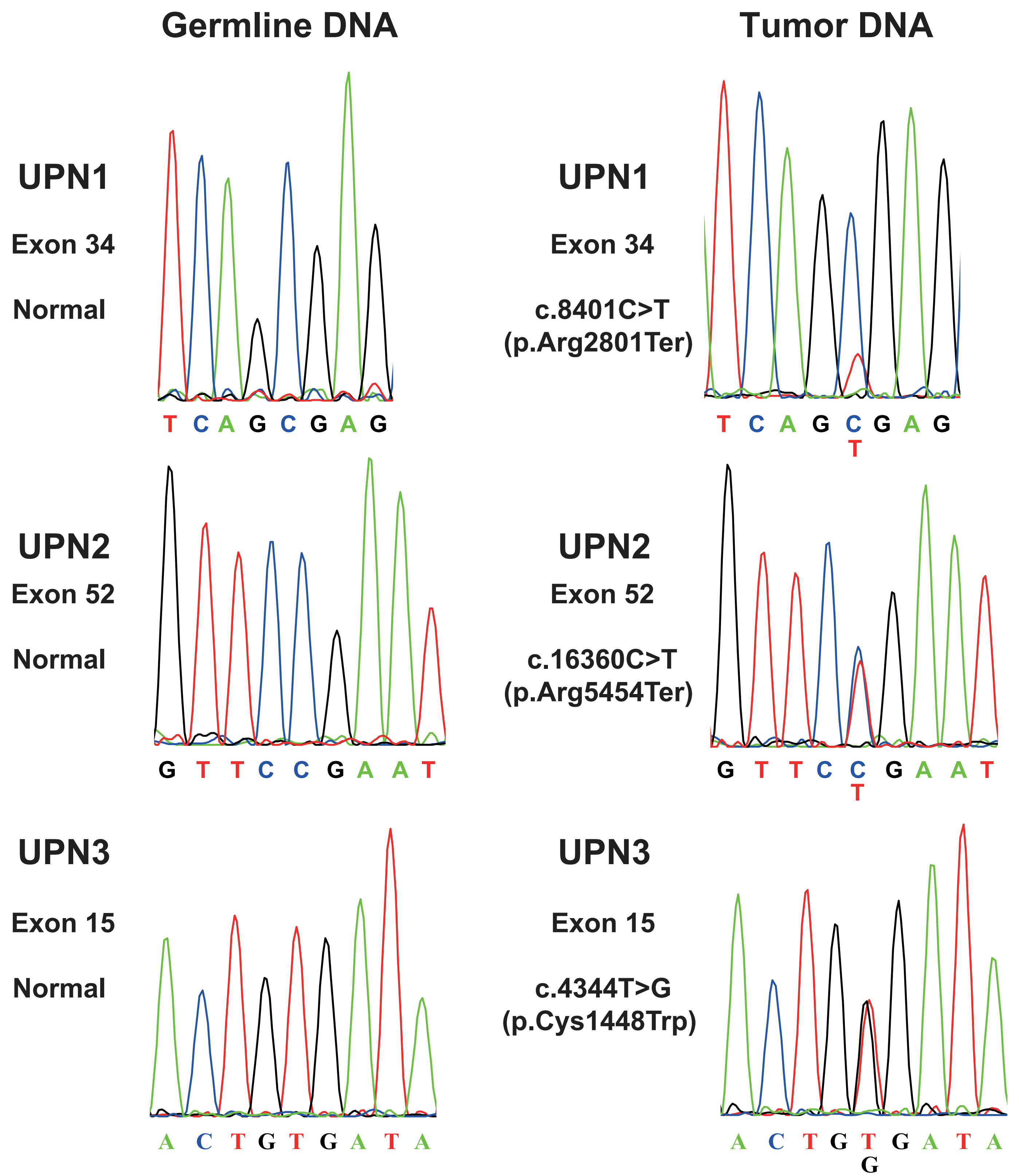

Figure S1. Results of sanger sequencing of 3 cases with *KMT2D* variants

Left side is germline DNA and right side is tumor DNA. UPN1 and UPN2 were nonsense variants and UPN3 was a missense variant.The mutation allele frequencies of three cases by whole exome sequencing analysis were 21%, 49% and 43% respectively. The reference sequence was NM_003482.

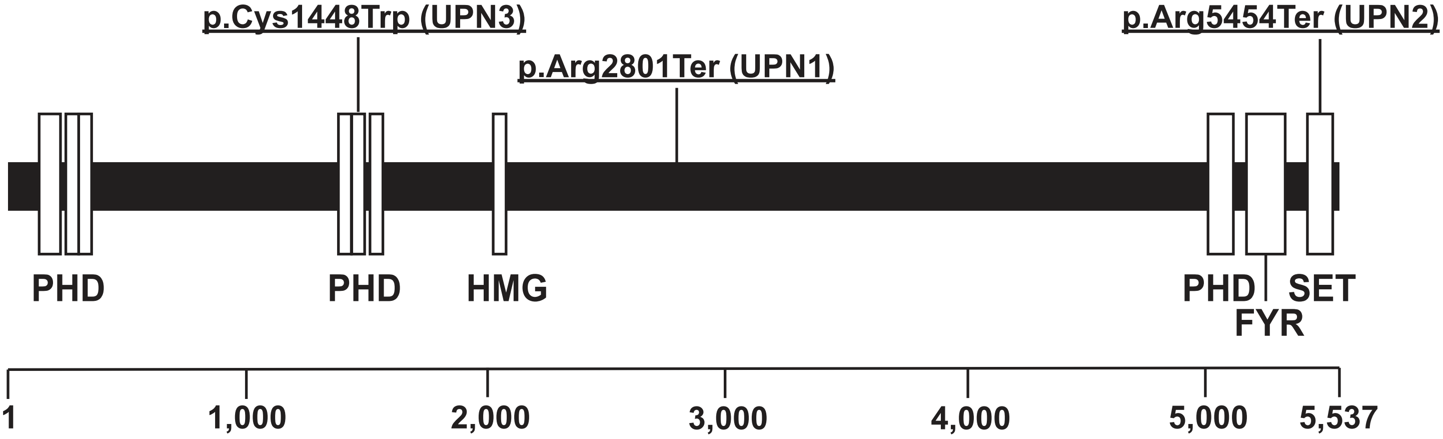

Figure S2. Schematic representation of the KMT2D protein and mutational diagram of 3 cases with *KMT2D* variants

PHD, plant homeodomain; HMG, High mobility group domain; FYR, FY-rich domain; SET, Su (var)3-9 Enhancer of zeste and Trithorax domain. The reference sequence was NP_003473.3.

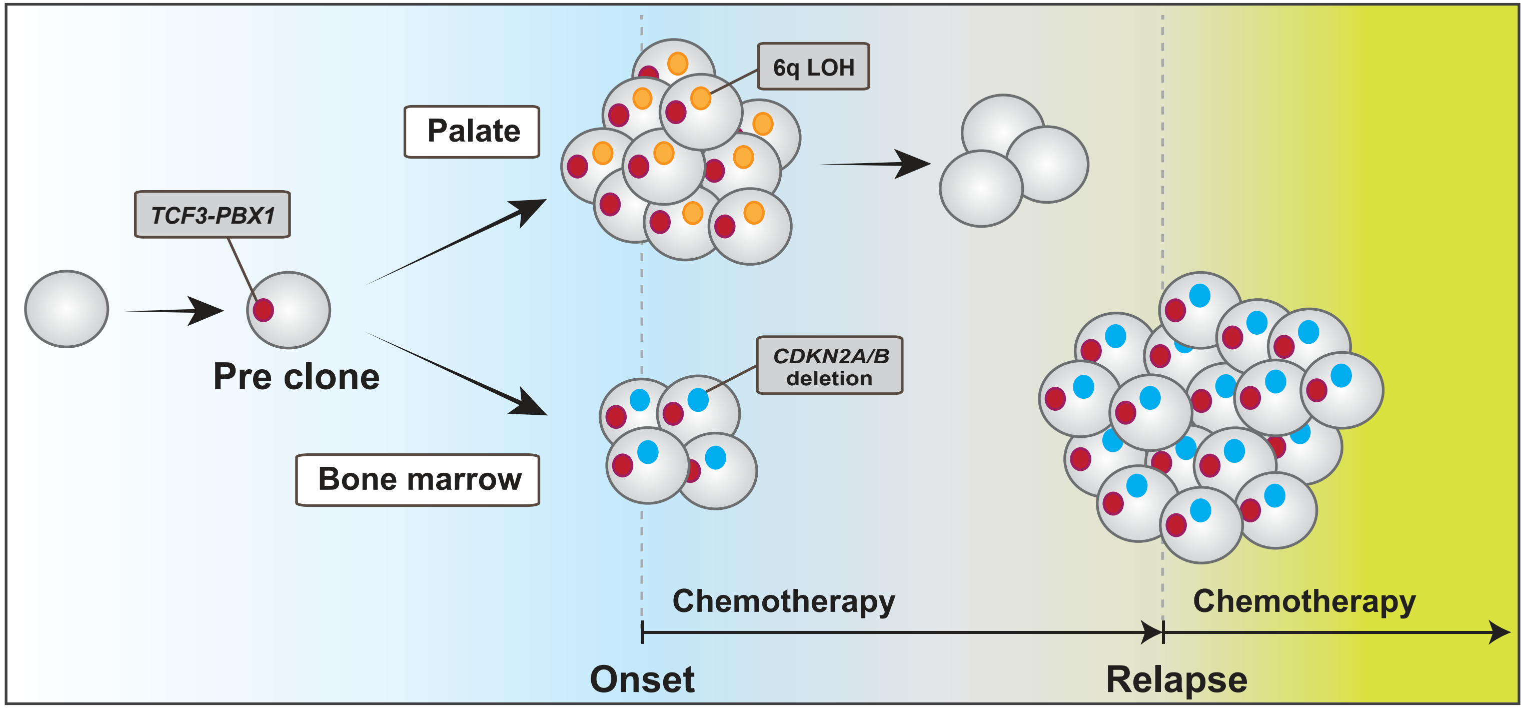

Figure S3. Schematic diagram of clonal evolution estimated from genetic analysis of recurrent case

TCF3-PBX1 was common to the palate and the bone marrow at diagnosis, but some genetic abnormalities were different including 6qLOH and *CDKN2A/B* deletion. Some genetic abnormalities including *CDKN2A/B* deletion in the relapsed clone were common to only a minor clone of the bone marrow at diagnosis.
